# Research progress of schema therapy for adolescents

**DOI:** 10.3389/fpsyg.2026.1810578

**Published:** 2026-04-13

**Authors:** Sicen Zhang, Lixia Zhang

**Affiliations:** 1 School of Public Health, Shaanxi University of Chinese Medicine, Xianyang, China; 2 Beijing Huilongguan Hospital, Beijing, China

**Keywords:** adolescents, clinical psychology, early maladaptive schemas, review, schema therapy

## Abstract

Adolescence is a vital time in human development, marked by fast physiological and mental changes that render teenagers more vulnerable to psychological suffering. With the growing frequency of mental health concerns among adolescents, the question of how to effectively intervene and help them in dealing with these challenges has emerged as a critical topic in the field of psychotherapy. Schema therapy, originally proposed by Young in the 1990s as an integrative psychotherapy approach, was initially developed for the treatment of chronic psychological disorders, particularly personality disorders. However, as schema therapy has evolved, its scope of application has gradually expanded, and numerous studies and clinical practices have begun to explore its use in adolescent populations. This paper focuses on schema therapy for adolescents, evaluating and summarizing important literature from 2000 to 2025 to inform future research and clinical practice.

## 1 Introduction

Schema therapy is a psychotherapeutic approach that integrates principles from cognitive-behavioral therapy, psychodynamic therapy, and Gestalt therapy into a unified model. The core concepts of schema therapy include early maladaptive schemas (EMSs), schema coping mechanisms, schema domains, and schema modes (Young et al., 2003). A schema refers to a cognitive structure developed through early life experiences, particularly those that are emotionally significant or traumatic (Skarnulyte et al., 2026). These schemas govern how individuals perceive and interpret their experiences, shaping their beliefs about themselves, others, and the world. In contrast, schema modes represent present-moment emotional-cognitive states that arise when a particular schema is activated in response to current emotional or environmental triggers. Schema modes differ from schemas in that they are state-like and reflect temporary emotional-cognitive responses, often accompanied by coping strategies (van Donzel et al., 2026).

It is crucial to distinguish between schemas and schema modes: schemas are relatively stable cognitive structures that shape an individual’s worldview over time, while schema modes are more fluid and describe the momentary activation of schemas, influencing emotional and behavioral responses in specific contexts (Piaget, 1952). This distinction is especially relevant in adolescence, as schemas formed in early childhood tend to persist throughout life, while schema modes reflect the dynamic nature of adolescents’ emotional states (Conway and Pleydell-Pearce, 2000). Piaget’s concept of cognitive schemas pertains to mental frameworks used to organize and understand knowledge, which, while related, are distinct from Young’s clinical schemas, which are more deeply rooted in emotional and relational experiences and arise from early-life adversity. Piagetian schemas pertain to knowledge organization, while clinical schemas in schema therapy reflect core beliefs about self-worth and interpersonal dynamics (Allé and Berntsen, 2026).

Schema Therapy was initially developed for individuals who did not respond well to traditional cognitive-behavioral therapy. These individuals often exhibit dysfunctional emotional and cognitive patterns originating from early adverse experiences, referred to as EMSs, which contribute to persistent maladaptive thought patterns and behavioral strategies. EMSs emerge from the interaction between temperamental traits, such as neuroticism and ongoing adverse experiences with primary attachment figures, such as parents, siblings, or peers (Geerdink et al., 2012). Young identified 18 distinct EMSs, which are categorized into five broad domains: disconnection and rejection, impaired autonomy and performance, impaired limits, other-directedness, and over-vigilance and inhibition. These domains provide a framework for understanding how early experiences shape an individual’s perception of self, relationships, and the world.

Schema coping mechanisms reflect how individuals respond to dysfunctional early experiences and EMSs (Vonk, 1999). These coping strategies are categorized into three primary types: overcompensation, avoidance, and surrender. Individuals engaging in overcompensation display behaviors that contradict their EMSs, such as striving to prove their self-worth in the face of schemas related to abandonment. Conversely, those engaging in avoidance attempt to avoid triggering situations that may activate their EMSs, while individuals who surrender yield to their schemas, behaving as though these beliefs are fact, thereby reinforcing the negative patterns associated with them (Conway and Pleydell-Pearce, 2000).

The role of schema modes in adolescent schema therapy is particularly salient, as adolescents experience heightened emotional reactivity and are more susceptible to schema activation. Schema modes represent temporary, state-like emotional-cognitive responses that are triggered in reaction to emotional stimuli, which can lead to rapid shifts in emotional and behavioral patterns (Rijkeboer and Huntjens, 2007). Understanding schema modes is vital in adolescent therapy because these states provide insight into how adolescents process emotional experiences and manage interpersonal challenges. For example, an adolescent with a disconnection and rejection schema may activate a vulnerable child mode when feeling neglected, or alternatively, enter an angry protector mode when confronted with perceived abandonment (Arntz, n.d.).

The development of schemas is intrinsically linked to adverse childhood experiences, which include neglect, trauma, victimization, overprotection, and selective internalization of dysfunctional family dynamics. These early experiences lay the foundation for the formation of maladaptive schemas (Lacey and Minnis, 2020). Adolescence represents a critical period for the development and consolidation of these schemas, as it is the time when individuals transition from childhood to adulthood (Dunn et al., 2011). During this period, adolescents face challenges related to identity formation, emotional independence, and the increasing complexity of moral and social decision-making (Thompson et al., 2012). As schemas become more entrenched in adolescence, they influence how individuals engage with their emotions, relationships, and coping mechanisms (Duke et al., 2010).

Research by Calvete et al. (2013) has highlighted that specific schema domains, such as those related to separation and rejection, autonomy and competence, and other-directedness are particularly prominent during adolescence. These domains align with key developmental tasks, such as emotional regulation, emotional independence, and the maturation of moral judgment. According to Erikson’s psychosocial development theory, adolescence is a time when individuals must navigate identity and role confusion in order to form a coherent sense of self (Erikson, 1968). As adolescents transition from concrete to abstract thinking, they gain the capacity to process more complex cognitive tasks. However, this cognitive shift also makes them prone to using simplified thought patterns when confronted with emotional or cognitive challenges. Consequently, EMSs are often activated and reinforced during this period, influencing adolescents’ emotional regulation and behavioral patterns (Linn and Songer, 1991).

## 2 Assessment of EMSs in adolescents

The fundamental goal of schema therapy is to identify a patient’s EMSs. Therapists utilize the Young Schema Questionnaire (YSQ) to determine which schemas need to be addressed and develop a therapy strategy (Young, 2005). With the advent of schema therapy, Young used factor analysis to modify the original questionnaire, resulting in the Young Schema Questionnaire - Short Form (YSQ-SF) (Young et al., 2003). Stallard and Rayner (2005) published the Schema Questionnaire for Adolescents (SQC) to examine adolescents’ EMSs. In their study, 47 adolescents aged 11 to 16 completed both the SQC and the YSQ-SF, and the results revealed that 10 of the 15 schemas in the SQC were strongly connected with the YSQ-SF outcomes. Güner (2017) created a new questionnaire to measure EMSs in adolescents using exploratory and confirmatory component analysis on a sample of 983 adolescents aged 10 to 16. The findings revealed that 14 schemas were congruent with Young’s EMSs, and had good fit indices, high internal consistency, and consistency over a 1-month period. Furthermore, it efficiently differentiated between clinically diagnosed and undiagnosed teenagers. Sijstermans et al. (2023) used a sample of 835 Dutch teenagers to give standardized data for the Dutch version of the Young Schema Questionnaire for teenagers, which was developed by Vlierberghe et al. (2004). The study’s conclusions included *Z*-scores, which allowed clinicians to determine whether an adolescent’s score was within the normal range.

## 3 Applications of schema therapy in adolescents

Adolescence is a critical developmental stage characterized by active identity formation, social reorientation, emotional reactivity, and increasing abstract cognition. During this period, adolescents are especially vulnerable to the formation of EMSs that can persist into adulthood (Geerdink et al., 2012). Schema therapy offers unique benefits by intervening early, before these schemas become deeply ingrained and consolidated. Adolescents’ schemas are still malleable, making this a prime window for therapeutic intervention to prevent chronic psychological issues in later life, such as anxiety, depression, and personality disorders. Early intervention thus helps reshape maladaptive cognitive patterns during a period of heightened cognitive and emotional plasticity, offering a significant opportunity to prevent the further entrenchment of problematic schemas (Dunn et al., 2011)^.^

To effectively treat adolescents, schema therapy has undergone several revisions to address the specific developmental demands of this age group. When conducting schema therapy with adolescents, it is crucial to adapt the therapy based on their individual cognitive and emotional developmental stages. Techniques such as visual rescripting, which help adolescents reimagine and process past negative experiences, must be tailored to suit the adolescent’s level of emotional and cognitive development. These adjustments ensure that adolescents can engage with therapeutic techniques in a manner that is appropriate to their stage of development, enhancing the overall therapeutic process.

In addition, adolescent schema therapy requires the active participation of parents or caregivers. The family plays a significant role in the establishment and reinforcement of EMSs, and including parents in therapy helps address family-based schema activation (Shahbazpour et al., 2026). This involvement creates a supportive therapeutic environment, promoting healthier family interactions that reinforce the adolescent’s recovery (Valente et al., 2026). Schema therapy can help identify and reshape maladaptive schemas at an early stage, preventing them from becoming deeply ingrained patterns that persist into adulthood. As a result, this approach allows for more effective intervention at a critical developmental juncture (Teymori et al., n.d.).

Furthermore, group schema therapy offers valuable benefits in creating an emotionally supportive environment where adolescents can engage in profound schema recognition and transformation. Adolescents in group settings can benefit from self-disclosure and observing others, which allows them to better understand their schemas and practice new interpersonal skills. This group dynamic promotes mutual recognition and experience sharing, which aids in emotional healing and fosters positive socialization. Adolescents participating in group schema therapy have reported enhanced interpersonal relationships, reduced feelings of isolation, and greater social connections, as highlighted by Monjezi et al. (2024).

The COVID-19 pandemic has significantly accelerated research into online schema therapy for adolescents (Feldman, 2022). Given that adolescents are often more comfortable with digital interactions, several researchers have explored the efficacy of online schema therapy. Preliminary data suggest that online schema therapy is effective for adolescents with emotional disorders (Stefan et al., 2023). Digital tools, such as interactive modules and virtual therapy courses, allow for a more adaptable approach to therapy, particularly for adolescents who face barriers to attending in-person sessions. This flexibility in delivery makes online schema therapy a viable option for increasing access to treatment, especially for those unable to attend face-to-face therapy due to geographic, logistical, or personal challenges.

## 4 Effectiveness of schema therapy in adolescents

The effectiveness of schema therapy in adolescents has been explored across various domains, including emotional disorders, personality disorder symptoms, behavioral problems, trauma, and eating disorders. While these studies provide promising evidence, a more systematic synthesis of outcomes and methodological quality is required to assess the true effectiveness of schema therapy in adolescent populations.

### 4.1 Adolescent emotional disorders

A number of studies have shown that EMSs can contribute to emotional disorders like depression and anxiety in adolescents (Tariq et al., 2021). EMSs related to emotional deprivation, abandonment, and defect are frequently activated during adolescence, often leading to the development of depression and anxiety symptoms (Cámara and Calvete, 2012). A systematic comparison of these studies, however, reveals that the strength of evidence varies: while Wainer et al. (2022) found significant reductions in anxiety and improvements in emotional regulation and self-esteem in adolescents receiving schema therapy, other studies focused more on individual conditions like disruptive mood dysregulation without direct comparisons to other therapeutic modalities (Abadeh et al., 2024). The lack of a unified measure of effectiveness across these studies hinders a clear understanding of schema therapy’s comparative effectiveness. The primary outcome indicators for emotional illnesses have been inconsistent. For example, while some studies show improvements in symptom reduction and emotional regulation, long-term maintenance and relapse prevention are rarely studied. Further standardization of success indicators, such as symptom reduction, emotional regulation, and functional outcomes across different contexts, may be advantageous in validating schema therapy’s overall efficacy in treating emotional disorders in adolescents.

### 4.2 Adolescent personality disorder symptoms

Schema therapy has been increasingly applied to adolescents exhibiting symptoms of personality disorders, particularly borderline personality disorder (BPD), given that adolescence is a critical period for the formation and consolidation of EMSs and coping strategies. Zamani conducted a study with adolescent girls who had BPD symptoms and discovered that schema therapy improved emotion regulation and interpersonal functioning by addressing maladaptive schemas like abandonment, emotional deprivation, and mistrust. Although these findings are intriguing, the study did not use a randomized controlled design, and the sample size was rather small, limiting the generalizability of the data (Zamani et al., 2021). In addition to BPD, schema therapy has been used to treat teenagers suffering from social anxiety and avoidant personality disorder. Baljé et al. (2024) explored the effects of group schema therapy on adolescents with these problems, focusing on social isolation and maladaptive interpersonal tendencies. The intervention provided measurable increases in social confidence and reduced avoidance tendencies, allowing participants to engage more actively in social and academic areas (Baljé et al., 2024). Collectively, these studies suggest that schema therapy may be effective in addressing a range of personality-related difficulties in adolescence by promoting adaptive emotion regulation and interpersonal functioning.

Despite these hopeful findings, the evidentiary base remains varied. Variations in study design, sample characteristics, treatment format, and outcome measures make direct comparisons difficult and prevent definitive conclusions about the relative efficacy of schema therapy versus established interventions such as dialectical behavior therapy (DBT), which is commonly used for BPD. Future research should use rigorously controlled, adequately powered trials to systematically evaluate schema therapy across various personality disorder presentations, investigate the durability of treatment effects, and determine the mechanisms by which schema-focused interventions exert their therapeutic impact in adolescent populations.

### 4.3 Adolescent behavioral problems

Disruptive behavior in adolescents is often linked to EMSs, such as mistrust and emotional deprivation (Van Wijk-Herbrink et al., 2017). A number of research, like Ramshe’s, have shown that schema therapy can enhance anger control and prosocial behavior by addressing the underlying schemas. However, there is very little systematic comparison between research. Ramshe’s study indicated favorable changes in emotional regulation in teenagers who self-harm, however the sample size was tiny and there was no comparison group (Ramshe et al., 2022).

A more rigorous review of existing research could help determine whether schema therapy improves anger management, self-harm reduction, and impulse control. While individual studies show promising effects, the variety of outcomes, such as self-harm, aggression, and general behavior, necessitates a better knowledge of which specific behavioral difficulties respond best to schema therapy.

### 4.4 Adolescent trauma issues

Several studies have explored how schema therapy can address trauma-related EMSs, such as mistrust and emotional deprivation. Tashkeh et al. (2019) found that schema therapy was effective in improving social acceptability and reducing bullying behavior in adolescents exposed to trauma. However, this study did not specify technical adaptations made for trauma processing, such as imagery rescripting and other experiential techniques, making it difficult to assess whether these techniques were used consistently or how they contributed to the overall success of the intervention (Tashkeh et al., 2019).

There is conflicting information regarding how trauma-focused variations of schema therapy compare to traditional trauma therapies. The emergence of conflicting data on trauma treatment necessitates further rigorous research into the technical changes required for properly implementing schema therapy in trauma cases. Further research should focus on the role of imagery rescripting and other experiential strategies in trauma processing in adolescents.

### 4.5 Adolescent eating disorders

EMSs related to emotional deprivation, self-criticism, and lack of self-discipline are frequently observed in adolescents with eating disorders, including binge-eating disorder and anorexia nervosa (Zhu et al., 2016). Empirical evidence suggests that schema therapy can positively influence eating attitudes, body image, and emotional regulation, resulting in measurable reductions in disordered eating behaviors (Rasouli Saravi et al., 2019). Moloodi et al. (2010) demonstrated that adolescents with binge-eating tendencies exhibited improvements in EMS-related cognitions and eating behaviors following schema therapy, while Sharifi and Etemadi (2012) found that group schema therapy effectively reduced eating disorder symptoms and associated shame in adolescent females with anorexia nervosa. These findings indicate the potential utility of schema therapy for targeting core cognitive-emotional patterns that contribute to eating pathology.

However, the generalizability of these findings is restricted. Many studies focus on specific eating disorder subtypes, use limited sample numbers, or rely on short-term follow-up assessments, limiting the capacity to draw conclusions about larger adolescent populations and the long-term efficacy of therapy. Furthermore, the variability of interventions—including individual vs. group forms, session frequency, and family engagement integration—makes cross-study comparisons difficult. Future research should focus on robust, large-scale, randomized controlled trials with long follow-up periods to assess the efficacy of schema treatment in encouraging long-term maintenance and relapse prevention in teenage eating disorders. Comparative studies examining schema therapy in relation to other evidence-based interventions, such as cognitive-behavioral therapy for eating disorders or family-based therapy, are also required to determine whether schema therapy has unique or additive benefits in addressing the cognitive-emotional mechanisms underlying disordered eating. Additionally, studies should examine the mechanisms of change, including which specific EMSs are most responsive to intervention, and how modifications in schema-related cognitions translate into sustained behavioral improvements.

In conclusion, schema therapy offers significant promise for treating a range of psychological problems in adolescents, but further research is needed to establish its comparative effectiveness, optimal treatment formats, and long-term impact. Future studies should focus on addressing the methodological gaps identified and refining the technical aspects of schema therapy to ensure its wide applicability and effectiveness in this population.

## 5 Advantages and limitations of schema therapy for adolescents

Schema therapy shows several potential advantages for adolescent mental health interventions, particularly because it offers an integrative framework that links cognitive content, emotional experience, and behavioral coping. First, schema therapy targets EMSs and their associated coping styles and mode activations, which may help clinicians explain and intervene in the co-occurrence of emotional distress, interpersonal sensitivity, and maladaptive behaviors often observed in adolescence (Antonini, 2022). Unlike symptom-focused techniques alone, this mechanism-oriented perspective can provide a coherent case formulation that connects early experiences, current triggers, and repeating patterns of reaction (Di Vincenzo et al., 2025). Second, adolescence is a developmental stage in which self-concept, emotion control abilities, and interpersonal orientation are still forming. From a developmental perspective, intervening before schemas and coping mechanisms become more rigid may provide a window for early adjustment, with possible preventative benefit for later chronic psychopathology (Bär et al., 2023). In addition, schema therapy is well-positioned to incorporate contextual influences that are especially salient for adolescents. Many adolescents’ schema activations occur within family interactions and peer contexts; therefore, schema therapy’s emphasis on identifying triggering environments and involving caregivers may support generalization of treatment gains to daily life settings (Edwards, 2022).

Despite these strengths, several limitations should be acknowledged. A key challenge concerns developmental fit and treatment delivery. Adolescents differ substantially in abstract reasoning, metacognitive capacity, and emotion labeling skills, and some schema therapy techniques particularly more complex experiential procedures may require careful developmental adaptation to avoid mismatch between emotional activation and integration (Joshua et al., 2025). Another challenge involves the family system. While caregiver involvement can enhance consolidation and reduce ongoing schema reinforcement, it may also introduce practical and clinical difficulties in high-conflict families. Thus, clearer guidance is needed regarding indications, level of caregiver involvement, and implementation strategies across different family contexts (Yousefi, 2011).

Finally, the current evidence base for adolescent schema therapy remains limited and heterogeneous. Existing studies differ in design quality, sample characteristics, delivery formats, treatment dose, and follow-up duration, which complicates cross-study comparison and reduces certainty about generalizability across settings and cultures. Therefore, conclusions about effectiveness should be framed cautiously, and future reviews should explicitly consider evidence strength and methodological limitations when synthesizing findings.

## 6 Conclusion and future outlook

In summary, schema therapy has promising relevance for adolescent psychological interventions, particularly for difficulties involving persistent negative self-beliefs, emotion regulation problems, interpersonal distress, and maladaptive coping patterns. By focusing on EMSs, coping styles, and mode activations, schema therapy may not only reduce current symptoms but also contribute to improvements in functioning (Karimipour et al., 2021). However, the existing research lacks sufficient consistency and high-quality evidence to support broad claims of comprehensive effectiveness across adolescent populations and issue categories.

Future research and clinical development may benefit from several directions. First, stronger empirical designs are needed, including well-powered randomized controlled trials or robust controlled studies with transparent reporting of treatment dose, therapist training, fidelity, attrition, and follow-up outcomes (Masley et al., 2012). Second, clearer delineation across intervention kinds would help with evidence synthesis. Studies that employ related but unique methodologies should be properly classified to avoid overgeneralizing outcomes under a single label. Third, future research should look more comprehensively at developmental and technical adaptations for teenage schema therapy. This involves outlining how experiential procedures are varied by age and developmental stage, the structure of caregiver modules, and how treatment intensity and format influence outcomes (Esteki Organi and Gorji, 2023). Fourth, a broader outcome assessment is recommended. In addition to symptom change, research should incorporate measures of emotion regulation, interpersonal functioning, identity-related outcomes, and maintenance indicators to better capture the developmental goals of adolescent therapies. Finally, cultural adaptation demands more attention. Because schema content, emotional expression standards, and family structures vary across cultural contexts, cross-cultural validation is required to allow responsible dissemination and implementation (Zhang et al., 2025).

As adolescent mental health needs increase, schema therapy may become an important intervention choice. Progress in this area will be dependent on improved evidence synthesis, more conceptual accuracy, and more explicit developmental and cultural customizing based on empirical research.
